# Supplementary material for: Are carbon emissions trading and green financial instruments synergistic? -Comprehensive quantitative research based on content analysis
Source: PLoS One. 2024 Mar 7;19(3):e0298601. doi: 10.1371/journal.pone.0298601 (PMC10919601; doi:10.1371/journal.pone.0298601)
Supplement: S1 Data — (ZIP) [file pone.0298601.s001.zip › Data/地级市Z_F工作报告.zip!/║╙▒▒/▒ú╢¿╩╨/└┤╫╘╣1⁄2╓┌║┼í╛┬φ┐╦╩2╛▌═°í┐_2019.docx]

各位代表：

现在，我代表保定市人民政府，向大会报告工作，请予审议，并请各位市政协委员和列席会议的同志提出意见。

一、2018年工作回顾

过去的一年，是全面贯彻党的十九大精神开局之年，也是保定经济社会发展不平凡的一年。在省委、省政府和市委的正确领导下，我们深入学习贯彻习近平新时代中国特色社会主义思想和党的十九大精神，抢抓京津冀协同发展和雄安新区规划建设重大历史机遇，围绕“十个紧紧抓住”的要求，坚持稳中求进总基调，统筹推进稳增长、促改革、调结构、惠民生、防风险各项工作，较好地完成了市十五届人大三次会议确定的目标任务，在决胜全面建成小康社会的征程上又迈出了坚实一步。全市生产总值3070.9亿元、增长7%（不含雄安三县，下同），地方一般公共预算收入256.7亿元、增长11.4%，固定资产投资增长5.9%，实际利用外资增长25.7%，社会消费品零售总额增长9.1%，规模以上工业增加值增长5.1%，城乡居民人均可支配收入分别增长8.7%、10.4%，呈现出总体平稳、稳中有进、稳中向好的发展态势。

一年来，我们认真贯彻落实市委的决策部署，强势起步、实干开路，主要做了以下几个方面的工作。

（一）持续推进京津冀协同发展，借势借力雄安新区，重点项目建设进一步加快。以中央公布实施河北雄安新区规划纲要为契机，谋划实施省市重点项目401项，完成投资661.6亿元，其中省重点项目个数和规模位居全省前列，全省项目观摩调度会在我市召开。优势产业项目加快聚集，中国信息通信研究院保定分院、新疆特变电工等重大项目相继落户，深圳华讯方舟太赫兹产业基地、深圳光启创新产业基地、中船重工涿州海洋装备产业园、河北京车满城轨道交通产业园、中电科涞水产业园、北京公交智造产业园等一批“中”字头、“京”字头、“深”字头项目加快推进，全面协同、深度融合的发展格局加快形成。我市被世界著名品牌大会评为“2018年中国最具投资潜力城市50强”。

（二）持续推进转型升级，调整优化产业结构，经济发展质量进一步变好。深入推进供给侧结构性改革，坚决去、主动调、加快转，印发了推进高质量发展的实施意见，制定了产业规划，明晰了发展路径。拆除曲阳田原热力、大唐华源热电机组，去除火电产能28万千瓦；关停曲阳太行山水泥厂，去除产能80万吨。深入推进“千企转型”，争列省技术改造项目114个，实施重大技术改造工程120个。深入推进战略性新兴产业发展，高新技术产业投资增长35.9%，规模以上高新技术产业增加值增长17.4%、占规上工业增加值53%，远超全省的平均水平。深入推进现代农业园区高质量发展，粮食丰产丰收，“粮改饲”取得新突破，认定“三品一标”农产品279个、无公害绿色生产企业309家。深入推进现代服务业快速发展，建设全域旅游示范区，成功举办第二届旅发大会，全市接待国内外游客1.12亿人次，旅游总收入1198亿元、增长25%；发展快递物流业，快递业务量突破3.5亿件、增长43%，位列全省第2位，超过山西、吉林等10个省份快递业务量；工业设计、专业技术、软件和信息技术等服务业营业收入增长10%以上，长城汽车获“2018年中国优秀工业设计奖”金奖。全市服务业增加值增长10.1%、贡献率61.7%，三次产业结构由上年的10.3∶45∶44.7调整为10.5∶41.6∶47.9，经济结构变轻变绿，发展质量越来越好。

（三）持续推进协同创新，加快新旧动能转换，内生发展动力进一步增强。深入实施创新驱动战略，扎实推进石保廊全面创新改革试验区和京南科技成果转移转化示范区建设，加强与中科院、中科协、中关村、深圳市的战略合作。保定·中关村创新中心和产业基地入驻企业227家，中关村智造大街保定创新中心顺利落地，中关村满城科创基地顺利推进，“一中心一基地多园区”格局加快形成。加强与深圳全方位合作，全力打造“1＋3”合作模式，深圳园展示中心投入使用，创投中心开工建设，120家企业准备入园，深圳湾科创基地双子楼竣工，深安智谷园区签约企业35家。加强与百度公司合作共建智能交通示范城市，工信部自动驾驶封闭测试场在大王店建成挂牌。高新技术企业新增300家、达到739家，科技型中小企业新增1000家、达到7517家，研发经费投入强度连续两年全省第一。

（四）持续推进生态建设，铁腕治霾强力治污，空气水体环境进一步好转。坚守生态底线，推动绿色发展，单位生产总值能耗降低率完成省定目标。深入落实减煤、抑尘、“散乱污”企业治理等综合管控措施，完成气代煤、电代煤改造23.9万户，关停整治“散乱污”企业1443家，全市PM2.5平均浓度下降20.2%，改善幅度全省最大。全面推进河长制，深入开展白洋淀流域治理十大专项行动，整治纳污坑塘1424个、清理河道垃圾145万方、封堵非法排污口211处、实施生态补水6.5亿方，河流断面水质总体达标率90%，多年没水的瀑河水库重新蓄水，多年干涸的瀑河、南拒马河开始清水常流。推进“一核三带八廊十片林、五库九河十湿地”生态体系建设，完成造林绿化72万亩，森林覆盖率达到32.2%，生态环境越来越好，绿地蓝天白云越来越多。

（五）持续推进精准脱贫，聚焦打硬仗补短板，贫困群众生活进一步改善。实施脱贫攻坚三年行动计划，划分四大战区，强化“十个再聚焦”，推进“七个全覆盖”。加大对深度贫困县、贫困村扶持力度，选派驻村干部、第一书记3250名。以“两不愁、三保障”为重点，整合涉农资金19.3亿元，市本级财政投入专项扶贫资金1.47亿元、同比增长31.6%。新放扶贫小额贷款1.21亿元，惠及贫困户2598户。新建光伏扶贫电站313个，达到511个、516兆瓦，3.9万贫困户受益。培育股份合作制的种养加扶贫龙头企业175家，发展现代农业园区112家，涉及贫困村535个，直接吸纳2.1万贫困人口就业。新增“扶贫车间”486个，贫困村覆盖率达到80%以上。深化拓展“太行山道路”，建成太行山农业创新驿站30个、实验推广基地8.6万亩，辐射带动7万农户增收。深化京保扶贫协作，完成对口帮扶项目183个，投入资金26.9亿元。9个贫困县的农村公路开工建设941公里、完工755公里，228个贫困村受益。易地扶贫搬迁贫困人口3.6万人，2080户危房改造基本竣工。全市347家定点医疗机构全部实现“先诊疗后付费”“一站式结算”。集中救治患大病贫困人口6100人、救治率99.6%，重病贫困人口兜底救治率达到100%。雨露计划补助9610人，做到了应补尽补、应助尽助。全市7.1万名建档立卡贫困人口实现脱贫，易县、博野脱贫摘帽，顺平、唐县、曲阳、涞水通过省脱贫退出验收评估。

（六）持续推进城乡建设，着力理秩序解难题，城市形象进一步提升。出台《关于进一步加强城乡规划工作的决定》，编制了保定全域“规划树”。坚持“房住不炒”，实行“双限双竞”出让模式，房地产市场秩序逐步好转。持续开展拆违风暴，坚决打击违法占地、违法建设行为，整改违法用地1万多亩，拆除违法建设1100多万平方米，拆除广告塔牌4555处、各类违法违规广告3.7万多块，“不敢违”的态势持续巩固。着力改善居住条件，改造棚户区住房46064套，改善老旧小区133个，解决房地产开发历史遗留问题567个。努力解决出行难问题，改造完成7条46公里城市主次干道，有效解决了雨污分流、强电弱电入地等短板问题；打通瑞兴路、李庄街等6条“断头路”，瑞祥大街北延、白洋淀大道最后一公里建成通车，双源无轨电车开通运营；火车站东广场、高铁站地下停车场投入使用，群众出行更方便；着眼融入“轨道上的京津冀”，谋划推进雄保忻、雄石等铁路项目，积极构筑全国性铁路交通枢纽；曲港、荣乌、涞曲、西阜高速建成通车，完成了“四纵四横两环”高速公路网建设，实现了县县通高速；新建改建农村公路1084公里，建成“四好农村路”省级示范县7个，易县被命名为全国示范县。着力弥补城乡建设短板，改造供热管网23.4公里，新建改建城市公厕136座，新建改建竞秀公园、宪法主题公园等23个公园游园，创建省级园林式单位、小区、街道16个，涞源省级园林县城创建成功，市区通过了国家园林城市复检。关汉卿大剧院投入使用，东湖文化中心的建设进一步加快。21个县（市、区）实现农村生活垃圾清理市场化，4826个村推广城乡一体化处理模式，农村人居环境越来越整洁。这里还需要特别报告的是，在城市集中供热管网改造基本完成的基础上，去年我们集中开工建设了一批道路改造提升工程，给市民出行带来不便，人大代表、政协委员和广大群众给予了充分理解和大力支持，在此深表谢意。

（七）持续推进“放管服”改革，深化“双创双服”，营商环境进一步优化。承接省重点改革任务172项，实施市改革任务181项，顺利完成市级机构改革任务，县级机构改革有序推进。加快“放管服”改革，打造“两个代办”升级版。深化行政审批局改革，完成市县行政审批局职能承接和规范运行，建立了“一枚印章管审批”的服务新机制。加快审批职能深度融合，推进审批流程再造，清理规范审批服务事项142项，减少审批环节133个。积极推动“一网、一门、一次”改革，开展审批服务事项“只跑一次”和“减证便民”专项行动，企业开办时间由8个工作日压缩至5个工作日，群众和企业办事创业更方便。围绕“创新、强企、解难、惠民”主题，优化政务、法治、金融等十方面环境，建立领导干部包联企业、清单管理、问题解决定期报告等六项制度，对992家企业和390个项目实行常态化精准帮扶。落实减税降费政策，减免企业税费33.4亿元。各类市场主体新增8万户、达到63.3万户。全市新增挂牌上市企业9家、达到104家。全市各项存款余额6887亿元，贷款余额4014亿元，存贷比58.29%、比年初增加5.29个百分点，增量存贷比105%、同比提高9.8个百分点，金融支撑力进一步增强。

（八）持续推进社会事业发展，着力保障改善民生，人民群众幸福指数进一步提高。全年用于保障和改善民生的财政支出557亿元，占总支出的83.6%。去年确定的47项惠民实事完成44项，其它3项正在抓紧推进。扎实推进城市公立医院改革，在全省率先建立了市互联网医院和县乡村“三级一体”医疗服务代办体系，唐县人民医院“一站式结算综合服务”被国家卫健委评为改善医疗服务活动十大亮点奖。实施中心城区和县城学校建设两个“三年攻坚行动”，新建改扩建学校77所。推动义务教育均衡发展，蠡县、阜平、涞源、顺平等10个县通过国家评估，提前两年实现县域全覆盖。深入开展安全生产大排查大整治行动，没有发生重大以上安全事故。坚决打好非洲猪瘟防控阻击战，食品药品安全形势平稳向好。积极防范化解金融风险，重大非法集资案件处置取得重大突破。强力推进扫黑除恶专项斗争，成功打掉涉黑组织8个、涉恶团伙82个，抓获各类犯罪嫌疑人1501人，社会治安不断好转，社会大局持续稳定。文化事业得到新发展，体育强市建设取得新成果，农村大喇叭工程成为传递党和政府声音的新阵地，社会主义核心价值观深入人心，全国文明城市创建迈出新步伐。退役军人服务管理工作扎实推进，国防动员和双拥共建深入开展。全国地方志工作经验交流会在我市召开。审计统计、民族宗教、新闻出版广电、档案、外事侨务、人民防空、气象、防灾减灾、妇女儿童、老龄、残疾人等工作都取得新成绩。

（九）持续推进政府自身建设，依法行政阳光行政，政务服务能力进一步增强。始终把加强政治建设摆在首位，牢固树立“四个意识”，坚定“四个自信”，坚决做到“两个维护”。自觉强化党组“负总责”的主体责任意识，严肃党内政治生活。对中央和省委巡视、国家和省扶贫考核、中央环保督察、中央扫黑除恶专项督导、省大督查组等多方面的反馈意见，精心制定整改方案和责任清单，按时保质完成整改任务。狠抓反腐倡廉，把行政权力关进制度的笼子，严格落实“一岗双责”，驰而不息纠“四风”，建立“问责＋清理”长效机制，加强督促检查和重点工作大督查，机关作风明显好转。认真学习贯彻《宪法》，不断增强依法行政能力，自觉接受人大法律监督、政协民主监督和社会监督。围绕《保定市白洋淀上游生态环境保护条例》《保定市中小学校幼儿园规划建设条例》两项地方立法，积极做好相关工作。办理人大代表建议82件、政协提案351件，见面率、办结率、答复率、满意率100%，受到省通报表扬。

各位代表，这些成绩的取得，是市委总揽全局、正确领导的结果，是市人大、市政协有效监督和大力支持的结果，是广大干部群众团结协作、不懈奋斗的结果。在此，我代表市人民政府，向全市人民，向人大代表、政协委员，向各民主党派、工商联、各人民团体和社会各界人士，向老干部，向各位企业家，向驻保单位、解放军指战员、武警官兵、政法干警和应急救援人员，向关心支持保定发展的朋友们，致以诚挚的敬意和衷心的感谢！

我们也清醒地认识到，经济发展还有不少风险和挑战，政府工作还有不少问题和缺点，群众生活还有不少操心事和烦心事。一是经济下行压力仍然较大，停产半停产企业较多，新旧动能转换仍处于爬坡过坎阶段，长期存在的深层次矛盾还没有得到有效解决。二是县域经济依然薄弱，加快发展的压力依然很大，发展质量水平与推动京津保地区率先联动发展目标还有较大差距。三是传统优势产业支撑作用减弱，新能源及输变电、纺织服装、建材等三大主导产业处于下降区间，新一代信息技术、生物医药等战略性新兴产业虽然快速增长，但规模偏小、支撑力不足。四是城市基础设施建设历史欠账较多，教育、医疗、养老等公共服务还有不少短板，新老城区融合发展步伐不快。五是大气污染治理的效果与群众的期盼还有差距，统筹协调好群众生活、企业生产和雾霾治理还有许多工作要做。六是政府个别部门和工作人员思想还不够解放、能力还不够适应、作风还不够扎实、干事创业的劲头还不足，工作效率还不高、服务意识还不强，等等。这些问题，我们一定会高度重视，不掩盖、不回避，采取有力措施，认真加以解决。

二、2019年政府工作的总体思路和目标任务

今年，是中华人民共和国成立70周年，是全面建成小康社会的关键一年。中央经济工作会议指出，我国发展仍处于并将长期处于重要战略机遇期。加快经济结构优化升级、提升科技创新能力、深化改革开放、强化绿色发展、大力发展民营经济的政策措施落地实施，将给我们带来更多的发展机遇。今年，是京津冀协同发展国家战略深入实施的第五年，是雄安新区启动大规模建设的一年。雄安新区规划的公布实施，非首都功能疏解的步伐进一步加快，高端资源要素加速涌入，重大基础设施和生态环境工程全面开工建设，大量投资将在保定落地，我们的交通条件、生态环境将进一步改善，区位优势、战略地位将进一步凸显，投资潜力、品牌魅力将进一步提升，必将让保定利好叠加。今年，更是我们抢抓机遇、加速奔跑最要劲的一年，是实现京津保地区率先联动发展大有作为的一年。经过我们持续不断地调结构，产业结构更趋合理；持续不断地推进科技创新，发展活力不断增强；持续不断地培育战略性新兴产业，质量效益不断提升。特别是雄安新区带来的“磁聚效应”，将为我们进一步加快结构调整、实现动能转换、推进高质量发展注入新的更强的动能。只要我们坚定不移地推动与雄安新区统筹发展、协调发展、错位发展、融合发展、一体化发展，做到思想上紧跟、行动上紧跟、规划上紧跟、产业上紧跟、发展上紧跟，保定跨越、崛起和复兴前景可待、未来可期！

各位代表！面对保定历史上从未有过的绝佳发展机遇，落实市委十一届六次全会精神，做好今年的政府工作，必须坚持“六个坚定不移”（坚定不移全面深化改革，坚定不移推动全方位扩大开放，坚定不移推动高质量发展，坚定不移落实以人民为中心的发展思想，坚定不移抓好环境建设，坚定不移加强党的领导），必须把握“三个聚焦”（聚焦改革开放，持续推进质量变革、效率变革、动力变革；聚焦协同发展，持续加快规划协同、产业协同、创新协同；聚焦攻坚克难，持续打好防范化解重大风险、精准脱贫、污染防治三大攻坚战），必须在“稳增长、提质量、增实力、重统筹、强保障”上下功夫（稳增长，就是坚持积极地稳、有效地投，落实“巩固、增强、提升、畅通”八字方针，千方百计确保经济平稳较快增长；提质量，就是坚持高标准地提、创新地提，不断增强发展源动力，推动经济高质量发展；增实力，就是坚持立足于新、立足于有，加速产业转型升级，做大做强民营经济，努力振兴实体经济；重统筹，就是坚持全域发展、可持续发展，牢固树立五大发展理念，统筹城乡一体化发展，统筹山水林田湖草系统治理；强保障，就是坚持聚焦聚力、高质高效，全面深化改革扩大开放，进一步优化营商环境，为经济社会发展保好驾护好航）。

今年政府工作的总体思路是：以习近平新时代中国特色社会主义思想为指导，深入贯彻党的十九大和中央、省、市系列会议精神，坚持稳中求进工作总基调，坚持新发展理念，坚持推进高质量发展，坚持以供给侧结构性改革为主线，落实“巩固、增强、提升、畅通”八字方针，坚持深化市场化改革、扩大高水平开放，大力实施“协同发展、创新驱动、环境支撑”三大主体战略，全力打好城市做大、产业做强、生态做美、民生做富四场攻坚硬仗，加快建设现代化经济体系，进一步稳就业、稳金融、稳外贸、稳外资、稳投资、稳预期，加快推动京津保地区率先联动发展，为全面建成小康社会收官打下决定性基础，以优异成绩庆祝中华人民共和国成立70周年。

今年经济社会发展的预期目标是：生产总值增长7%，一般公共预算收入增长6%，规模以上工业增加值增长5%以上，服务业增加值增长10%以上，固定资产投资增长7%左右，社会消费品零售总额增长9.5%，实际利用外资增长5%，出口总值保持正增长，城乡居民人均可支配收入分别增长7.5%和9%，居民消费价格涨幅控制在3%左右，其他约束性指标确保完成省定任务。这些目标，是本着积极稳妥、上下衔接、高处着眼、实处入手的原则确定的，总体上略高于全省平均水平，既考虑了国家宏观调控政策的拉动效应和一些不确定因素，又考虑了我市经济形势和支撑条件，体现了稳中求进和高质量发展的要求。

今年政府工作的主要任务是：紧紧围绕市委全会提出的“九个攻坚”要求，扎实做好九方面的重点工作。

（一）强力落实“巩固增强提升畅通”要求，千方百计稳增长。贯彻落实中央经济工作会议和省委九届八次全会、省两会精神，积极应对经济下行的巨大压力，集中力量抓投资、促消费、保运行、稳增长，力求稳中有进、持续向好。

狠抓项目投资。开展“重点项目建设质量年”活动，建立市、县重点项目储备库，实施三年滚动计划。坚持工作项目化、项目工程化，对2019年实施的项目，按照产业发展、城市建设、生态环保、农业农村、社会事业五大板块，分类实施，统筹推进。全市初步安排市以上产业类重点项目320项，年计划投资500亿元。实施重点项目审批程序简化专项攻坚行动，加快项目推进速度；实行项目扁平化管理，完善领导包联和清单管理制度，加强定期调度和现场办公，在要素配置上给予重点支持；健全督导、考核、奖惩机制，坚持一季一观摩、一季一测评，促进项目快开工、快建设、快达效。

积极扩大消费。实施全市旅游质量提升三年行动计划，推进文化和旅游深度融合发展，筹办好曲阜唐第三届全市旅游产业发展大会，发挥好西部山区高速公路开通后的旅游带动效应，加快发展全域旅游。实施现代商贸物流工程，建设一批交易额亿元以上的电子商务平台，加快河北新发地农副产品物流园二期、高碑店保税物流中心建设；推进快递物流园建设，构建覆盖城乡、便民惠民的快递骨干网和末端投递网，打造国家二级快递枢纽。实施重点商圈建设工程，重点抓好朝阳大街商圈、裕华路商圈，建设一批大型商业综合体，加快形成布局合理、多点结合的城市消费新格局。设立市级服务业发展引导资金，鼓励支持发展会展经济，鼓励支持定制消费、信息消费、假日消费、夜间消费，鼓励支持社会资本投向现代服务业，不断满足广大人民群众日益增长的差异化、个性化、多样化消费需求。落实个人所得税专项附加扣除政策，增加居民收入，增强消费能力。

强化运行调度。建立企业运行调度中心，将市县两级重点工业企业纳入全市工业运行监测平台，强化要素协调，在企业研发、创新、融资、用地、品牌等方面给予重点支持，保障大企业、好企业的水电气热供应。落实联系服务重点企业的“五个一”工作机制，“一企一策”帮助企业解决困难和问题。加强产需对接，围绕主导产业组织开展产业链上下游对接活动，帮助企业拓展市场。不搞环保“一刀切”，保证达标企业稳定有序生产。制定激励措施，促进“小升规”，指导符合入统条件的企业依法依规申报，全年新增入统工业企业100家以上。

放大政策效应。落实国家减税降费和金融支持实体经济的政策，降低社保缴费率，降低用能和物流成本，清理和规范各种附加收费，对小微企业和科技型初创企业实施普惠性税收减免。降低小微企业融资担保费率，持续开展政银企对接活动，引导银行向企业发放中长期贷款。鼓励企业通过挂牌上市、发行企业债、中期票据等方式融资。建立产业发展基金和小微企业应急转贷资金池，帮助企业破解融资难、还款难问题，探索解决融资贵问题。建立健全地方金融监管体系，严格防控金融风险，排查化解金融隐患，及时处置金融突发事件，防范打击非法集资行为。

（二）强力服务保障雄安新区建设，协同发展提质量。坚决服从、坚决支持、坚决借势、坚决保障雄安新区建设，着力打造国家创新型城市，增强高质量发展源动力。

在服务保障上用心用力。强化对雄安新区服务，坚决保障雄安新区的水、电、路等要素支撑，对雄安新区规划建设领导小组交办的事项，逐一建立台账，实行清单管理，第一时间落实到位。加快环雄安新区造林绿化、生态治理、郊野公园建设，推进白洋淀上游流域综合治理，为雄安新区提供强有力的环境支撑。深化与雄安新区常态化对接，加强对雄安周边协同发展区域的有效管理。做好后续托管移交工作，确保有序衔接。

在规划协同上对标对表。落实省委、省政府关于河北雄安新区总体规划的实施意见，全面对接雄安新区总体规划及白洋淀生态环境治理和保护规划，落实“五级两规一导则”要求，持续推进城市总体规划、城市设计、土地利用规划、综合交通规划、产业体系规划、白洋淀上游生态治理规划等专项规划编制。上半年编制完成雄安新区周边县总体规划，年底前编制完成县域总体规划、镇域总体规划、乡村规划，构建起多规合一的规划体系。

在交通建设上互联互通。从今年开始，雄安新区对外骨干路网项目将陆续启动，包括23条国省干线、5条高速公路，其中涉及我市国省干线16条段、高速公路3条段。今年重点推进安大线、容易线两条建材通道建设，加快推进津石高速、京雄高速、荣乌高速新线建设。统筹做好雄保忻铁路、雄石城际、廊涿城际、高铁东站综合交通枢纽规划建设准备工作。完成城市轨道交通线网规划，启动建设申报工作。保障好石家庄到雄安、张北到雄安特高压输变电工程建设。

在产业发展上对接承接。积极争取京津雄产业转移合作，优化承接环境，提高承接能力。制定实施保定全域对接雄安新区的产业转型升级规划和主导产业发展行动方案，加快构建以先进制造业、新能源和智能电网、新一代信息技术、生物医药健康、新材料和节能环保产业为主导的现代产业体系，力争通过两年努力，主营业务收入达到3500亿元。

在创新驱动上同频共振。深入实施创新驱动发展战略，用足用好石保廊全面创新改革试验区和京南国家科技成果转移转化示范区特殊政策，加快布局一批科技成果转移转化基地。进一步加强与中科院、中科协、中关村、深圳市等高端创新资源的战略合作，加快推进中关村“一中心一基地多园区”、深圳科创产业园、光启超材料产业园、华讯方舟太赫兹产业基地、同光晶体第三代半导体产业园、亦庄众联·保定创新园、中电科涞水产业园、保定（涿州）国家农业高新技术产业示范区等重点园区建设，形成创新产业聚集优势。做强创新平台，新增省级以上重点实验室2家、省级以上技术创新中心（工程技术研究中心）10家、产业技术研究院1家、院士工作站4家。继续实施科技倍增计划，加强科技型企业培育，新增国家级高新技术企业200家以上、科技型中小企业1000家以上。组织开展创新论坛，打造优质的创新创业环境，提高大众创业、万众创新水平。

（三）强力推进民营经济发展，壮大实体强实力。着力在产业抓特色、行业抓骨干、园区抓提升、服务抓改善上下功夫，大力发展民营经济，繁荣振兴实体经济。

做强战略性新兴产业。深入实施战略性新兴产业发展三年行动计划，抓好高技术产业化示范、创新百强培育等六大工程，打造新型产业链，推动产业聚集和技术转化，促进高新技术产业发展。加快特变电工科技创新产业园、长城汽车氢能产业示范园、河北京车满城轨道交通产业园、北京公交智造产业园等项目建设，力争主体工程年内完工或项目竣工投产。加强与百度、军事科学院、中关村智造大街、北控、首创等企业和科研院所的对接，在军民融合、科技成果转化上深度合作。积极推动松下电器、国鹰无人机航空产业园、中日循环经济产业示范园等项目落地。

做大行业龙头企业。全面深化与驻保央企的战略性合作，完善央企服务推进机制，精准优质高效服务。力促保定天鹅新型纤维制造一期、风帆储能蓄能项目竣工投产，加快推进中船重工涿州海洋装备产业园、乐凯新材料产业园等重点园区建设。积极争取央企在保设立或联合组建研究院所、实验室、新型研发机构、技术创新联盟等各类研发机构组织，催生新的经济增长点。扶持龙头企业做大做强，集中资源要素，支持长城汽车、奥润顺达、晨阳水漆等企业加快发展，支持荣毅通信光电网、隆泰丰博石墨烯、邦泰氨纶新材料等新产品新技术企业加速成长，支持四通新材并购重组立中集团整体上市，支持更多龙头骨干企业通过兼并、联合等形式，提高市场竞争能力和市场占有率，打造顶天立地的领军型骨干企业。

做优区域特色产业。毫不动摇支持民营经济发展，坚决落实各项政策措施，在市场准入、审批许可、招投标等方面一视同仁、公平竞争，为民营企业创造良好发展环境。每个县因地制宜选择明确1—2个特色产业,制定完善主导产业发展规划，做足特色产业文章。实施民营企业“百千万”提升工程，促进创新资源和先进要素向优质民营企业集中，引导中小企业向“专精特新”发展，培育铺天盖地的科技型中小企业。实施县域科技创新跃升计划，加快改造提升传统产业。传承发展中医药产业，推进科研、种植、加工、医疗、康养全产业链协同发展，打造安国中药都，建设中医药强市。以技术创新、标准引领和产业链条延伸为重点，高质量发展白沟箱包、高阳纺织、蠡县裘皮、曲阳石雕和定瓷、博野胶带、望都食品等一批县域特色产业集群，培育一批名牌名品，打造一批行业小巨人。

做实园区项目平台。完善园区发展规划，理顺体制机制，推进项目建设与开发区联动发展，促进园区提升能级和服务水平，集中打造一批定位清晰、功能齐全、产业聚集优势明显的特色产业承接平台。强化园区平台配套支撑，推进省级以上开发区尽快实现九通一平，重点园区有计划实现“双九通一平”。强化园区招商能力建设，夯实开发区“四个一”工作基础，推进精准招商。强化园区平台承载对接能力，围绕战略性新兴产业发展、传统产业转型升级和跨区域合作共建，重点培育一批试点示范园区。

（四）强力推进城市建设攻坚，提升品质出形象。把今年作为城建攻坚突破年，以“城市双修”为抓手，持续补短板、理秩序、提品质、促融合，彰显古城魅力、建设现代新城。

拉大城市框架。加快新三区与主城区融合发展，加快向阳大街、阳光大街北延项目建设，积极推进西二环北延、东三环北延、北三环西延、裕华路东延项目，确保复兴路西延、乐凯大街南延建成通车。优化中心城区布局，将学校、医院、体育场馆等公共服务设施逐步向新三区拓展，提升中心城市辐射能力。持续推进路网改造提升三年行动计划，改造提升阳光大街、复兴路、长城大街等9条市政道路，改善出行环境。同步推进排水防涝工程，强电弱电一同入地，加快补齐地下短板。

完善城市功能。推进城市基础设施建设，加快第二地表水厂、集中供热改造、环城天然气管道、鄂皖沧保定支线、定兴LNG储备站、鲁岗和银定庄污水处理厂扩能改造等工程建设。实施棚户区和城中村改造三年行动计划，继续实施老旧小区改造工程，鼓励支持有条件的住宅小区加装电梯。完成大世界商业城安置区拆迁任务，启动安置区建设。实施建华大街"城市双修"项目，打造“城市双修”示范工程。

规范城市管理。以创建全国文明城市为抓手，推进智慧城市建设，加强城市精细化管理。聚焦精扫细保、精整细治、精管细护、精疏细导，推进市容市貌“治脏”，马路市场“治乱”，市政设施“治差”，交通秩序“治堵”，努力管出秩序、管出形象、管出群众满意度。深化“一区三边”违法建设整治行动，严厉打击违法建设行为。深化违法占地专项整治行动，维护正常土地秩序。加强房地产市场管控，严厉打击非法销售行为，维护房地产市场稳定。实施园林绿化攻坚行动，抓好“三园、四带、四节点、十四游园”建设，让城市更绿更美，群众生活休闲更方便。

推进县城建设。落实省《关于加快县城建设推动县域经济高质量发展的实施方案（2018－2020年）》，瞄准打造高质量、高品位的“微中心”和特色城市，突出规划引领，深化产城融合，加快“七个一”和新“三个一”工程建设，增强承载能力，改善生态环境，补齐基础设施和公共服务短板，提升县城规模等级，统筹城乡一体化发展。

（五）强力实施乡村振兴战略，统筹发展惠“三农”。坚持农业农村优先发展，统筹推进产业、人才、文化、生态、组织振兴，让广大群众不断收获看得见、摸得着的实惠。

发展现代农业。推进农业供给侧结构性改革，对接京津、雄安市场，加快发展科技农业、绿色农业、品牌农业和质量农业。坚持藏粮于地、藏粮于技，实施高标准农田建设，确保粮食安全。调整优化种养结构，抓好肉、菜、蛋、奶等农产品生产。全面提升现代农业园区建设质量，力争省精品园区达到7个以上。持续创建农民合作示范社和示范家庭农场，培育新型农业经营主体，每个县（市、区）培育发展3到5个具有一定规模的农业生产性服务组织。创建农业技术服务平台，新建太行山农业创新驿站10家，农业信息服务平台覆盖率达到90%以上，主推技术与主导品种入户率不低于98%。

打造特色品牌。以做优质量、做出特色、做响品牌为方向，加快打造高品质有口碑的保定农业“金字招牌”。大力促进中药材、食用菌、马铃薯和饲草发展，建立涞源、涞水、阜平等西部山区中草药种植基地。实施农产品品牌创建工程和区域公用品牌培育工程，每个县至少打造1个农产品区域品牌、企业品牌、特色品牌。因地制宜发展资源开发型、资产经营型、休闲观光型集体经济，培育打造特色小镇，在西部贫困县开展“共享农舍”“共享农庄”“共享山庄”建设试点，探索乡村振兴新路径。

深化农村改革。深化农村土地制度改革，实现土地向经营大户、家庭农场、农民合作社、农业企业集中，流转率提高4个百分点、达到37%，规模化经营水平达到20%以上。深化农村集体产权制度改革，做好集体经济组织成员身份界定、股权量化等工作。实施强村富民工程，推动资源变资产、资金变股金、农民变股东。创新农业投融资机制，帮助龙头企业、创新驿站、农业园区解决融资难题，鼓励支持金融和社会资本更多投向农业农村。

加强乡村治理。按照城乡融合发展方向，落实“1＋5”乡村振兴规划体系，优化乡村空间布局。学习浙江“千村示范、万村整治”工程经验，扎实推进农村人居环境整治三年行动，以实施村庄清洁行动、农村垃圾处理、厕所革命、污水治理、村容村貌提升为重点，解决农村脏乱差问题。积极探索农村新型社区建设，规划管控新建农房，整治改造“空心村”，开展村街硬化、村庄绿化、街巷亮化，保护古树古木，传承乡土文化，培树文明新风，建设美丽乡村。

（六）强力打好脱贫攻坚战，决战决胜保出列。精准施策，精准发力，全市实现4万人以上贫困人口脱贫，确保阜平、涞源两县脱贫摘帽。

合力攻坚深度贫困地区脱贫。聚焦“两不愁三保障”，落实“精准”要求，下足绣花功夫，确保工作成效。抓实产业扶贫，不断提升特色农业、光伏、旅游、家庭手工业等扶贫产业的带动作用。支持有条件的贫困户将土地、林地、建筑物等生产资料折价入股专业合作社、龙头企业，让贫困户分享产业增值收益。推广“互联网＋扶贫”模式，支持曲阳、望都、顺平建设国家级电子商务进农村综合示范县。加强京保扶贫协作、“百企帮百村”等社会帮扶工作，加快项目资金落地见效，打造特色亮点。抓住教育保障，落实“两免一补”“三免一助”政策，为贫困县招聘400名特岗教师，发挥好“北京·燕太片区职业教育协作区”作用，加大职业教育和就业技能培训力度，对接京津冀用工单位，力争一人就业全家脱贫。抓好医疗和养老保障，落实建档立卡贫困人员“先诊疗后付费”“一站式结算”和医保报销“代办制”，为建档立卡、低保对象、特困人员等贫困人口每人每年代缴城乡居民养老保险费100元。抓死住房保障，完成1263户重点扶贫对象危房改造，年内完成易地扶贫搬迁任务，提升安置区基础设施和公共服务水平，确保搬迁贫困群众搬得出、稳得住、能脱贫。抓牢兜底保障，搞好农村低保制度与扶贫开发政策有效衔接，提高特困人口、贫困老人的集中供养（养老）覆盖率。抓紧基础配套，年底前所有贫困村要通硬化路、通动力电，户户通广播电视、通宽带，实现安全饮水全覆盖，相关配套公共服务达到脱贫出列标准。

精准推进非贫困县贫困人口脱贫。深入落实省《关于统筹推进非贫困县、非贫困村脱贫攻坚工作的指导意见》，加强非贫困县扶贫机构建设，在脱贫任务较重的乡（镇）设置扶贫工作站，贫困人口较多的非贫困村参照贫困村工作机制，安排专人负责扶贫脱贫工作。加大对贫困人口较多的非贫困村的资金投入和政策扶持力度，加快改善非贫困村的基础设施和基本公共服务，尽快让贫困群众甩掉“穷包袱”，过上好日子。

持续抓好脱贫成果巩固提升。完善防贫机制，开展扶贫扶志扶智行动，增强群众内生动力和自我发展能力。统筹做好“后三年”和“三年后”各项工作，对已经脱贫的县，包括非贫困县（市、区），强化动态监测、产业支撑、政策延续、保障兜底，让惠民政策之水，持续流进户里、洇进地里，不断提高脱贫质量。加大对贫困边缘户扶持力度，让他们的生活更殷实、日子更红火。

（七）强力推进污染防治攻坚，标本兼治优环境。更加突出水的治理，把白洋淀上游河流治理作为重中之重，当好京津和雄安新区的生态护城河，改善生态环境质量，提升保定生态魅力。

坚决打好碧水保卫战。按照河北省白洋淀生态治理和保护规划实施意见，严格落实白洋淀上游流域治理实施方案、河长制工作督查督办制度和巡河员管理办法，大力削减入河入淀污染负荷。实施水污染治理、水资源保护、水生态修复“三水共治”，全力抓好污水处理厂扩容提标、城镇雨污分流、黑臭水体和纳污坑塘整治、河道清理和湿地建设等重点工作，年底前消除城市黑臭水体，明年底前城市和县城生活污水收集率分别达到95%以上、90%以上，城市和县城生活垃圾无害化处理率都要达到98%以上。我们要以更坚定的决心、更大的工作力度，推进水污染治理，坚决不让一滴污水流进白洋淀。

持续打好蓝天保卫战。全面落实《打赢蓝天保卫战三年行动方案》和大气污染防治9项治本措施，持续实施燃煤锅炉改造提升等13个作战计划，力争PM2.5下降6%。坚持“以气定改、以电定改”，稳妥推进气（电）代煤，探索农村采暖新模式，按计划完成燃煤锅炉淘汰改造任务。加大扬尘、面源和移动源污染治理，严格管控秸秆焚烧，市区和县城禁放烟花爆竹，开展餐饮油烟和柴油货车污染专项治理。实施工业企业退城搬迁改造和燃煤电厂深度治理，开展挥发性有机物专项治理，“散乱污”企业持续动态清零。推进露天矿山综合整治，建设绿色矿山。打好净土保卫战，全面开展土壤污染状况详查，加大农用地、建设用地污染风险管控，严格危险废物监管，加强垃圾废弃物综合利用。开展生态环境大排查、大整治，坚守生态保护红线，以零容忍的态度严厉打击环境违法行为。

着力构筑绿色生态屏障。以创建国家森林城市为目标，大规模开展植树造林，全年完成造林65万亩，森林覆盖率达到33.8%。推进西大洋、王快、安格庄水库周边以及国有林场延展区、宜林山地规模化林场建设。持续推进“一核三带八廊十片林，五库九河十湿地”生态建设，重点实施廊道绿化、河道绿化、湿地恢复、水源涵养等十项工程，启动环雄安新区林带、郊野公园建设，努力打造天蓝、地绿、水清、景美的美丽保定。

（八）强力深化改革扩大开放，优化营商环境增活力。落实市委全面深化改革扩大开放的86条重要举措，着力优化营商服务环境，为高质量发展提供坚强保障。

深化重点领域改革。抓好市县机构改革后续工作，突出不同层级职责特点，合理界定市县各层级权力责任，使权责更加统一、监督更加有力、运行更加高效。调整完善市与区财政管理体制，继续深化经济发达镇行政管理体制改革，推进涿州市及松林店镇、白沟新城及白沟镇行政管理体制改革工作，探索新型城镇化与城乡统筹发展新路径。健全国有资产管理体制，推进市级经营性国有资产集中统一监管，促进国有资产保值增值，把国有资本做优做大。完善市属国有企业负责人经营业绩考核机制，促进市属国有企业良性发展。推广政府和社会资本合作模式，引导社会资本进入基础设施、医疗养老、教育文化、体育等重点领域。

深化“放管服”改革。推进市县乡村四级“互联网＋政务服务”平台建设，优化“一窗受理”“一章审批”“一站办理”“最多跑一次”服务机制，完成市民服务中心新址建设，让群众和企业进一个门、办所有事。深化与浪潮集团合作，构建“智慧政务”服务平台，建立行政服务大厅窗口服务质量电子评价系统，主动接受服务对象监督。完善市县电子交易公共服务系统，深入推进公共资源交易一体化建设和全程电子化建设。创新服务方式，学习借鉴沈阳、厦门改革经验和“拿地即开工”“一会三函”审批新模式，多图联审、多评合一、联合验收、容缺受理，开辟绿色通道，提高工程建设项目审批效率。落实国家市场准入负面清单制度，坚决打破“卷帘门”“玻璃门”“旋转门”。持续开展“双创双服”活动，构建亲清新型政商关系，给予企业家更多关爱和荣誉，依法保护企业家人身和财产安全，对有突出贡献的企业家大张旗鼓地表彰奖励，营造尊重关爱企业家的浓厚氛围。

扩大对外开放。强力推进精准招商引资，瞄准世界500强、跨国公司、研发机构和国内知名企业，加快引进一批战略投资者。主动融入“一带一路”建设，积极参与国际贸易合作，鼓励出口创汇，推动外贸转型升级。加强与德国、韩国、日本等国家开展国际友好城市交流合作，有针对性地组织企业参加国际国内会展。推动商贸流通加快发展，深化白沟市场采购贸易方式试点改革，支持标准化和智能化物流示范项目建设。加大招商引资工作考核力度，充分调动各方招商积极性。支持高碑店市办好第23届国际被动房大会和第五届中国国际门窗博览会，继续办好野三坡中国经济论坛。实施人才强市战略，构建“1＋N”人才招引政策体系，创建海外人才离岸创新创业基地，开设国际高端人才、高层次创新人才、产业紧缺人才引进绿色通道。推进在保高校创新人才中心建设，打造科技创新人才培养高地。

（九）强力推进社会事业发展，倾心尽力惠民生。从群众关心的事情做起，以群众满意为目标，大力增加基本公共服务有效供给，努力让群众有更多的获得感、幸福感和安全感。

补齐民生短板。持续深化医药卫生体制改革，加快市第一中心医院眼科大楼、市第一中医院、市二医院等项目建设。推进保定市互联网医院建设，力争年底实现全市二级以上网络医院全覆盖。全面促进教育事业均衡发展和质量提升，强力推进两个“三年攻坚行动”，新建改扩建中心城区学校18所、县城学校19所，培训中小学骨干教师3000人。大力发展职业教育，推动保定学院向应用技术类高校转型发展，加强职业院校“双师型”队伍建设，培养一批能工巧匠。

增进民生福祉。把稳就业摆在突出位置，重点解决好高校毕业生、农民工、退役军人等群体就业。加大援企稳岗力度，对连续缴纳失业保险费1年以上且无欠缴、不裁员或少裁员的参保企业，返还上年度实缴失业保险费的50%。加大农民工工资拖欠问题治理力度，落实保障义务教育阶段教师工资待遇的政策，解决好退役军人、下岗失业人员等群体合法利益保障问题。城乡居民医疗保险最高支付限额由15万元提高到20万元。推进社区和居家养老服务中心建设，构建以居家为基础、社区为依托、机构为补充、医养结合的养老服务体系。

丰富文化生活。加快淮军公所、清河道署、直隶审判厅修缮布展工作，打造特色文化街区。做好新博物馆展陈工作，加快推进市图书馆（档案馆）新馆建设，启动保定军校改造提升工程。开展“书香保定·香飘四季”系列活动，文化润古城惠民演出300场以上，继续办好“大激店世界音乐节”，举办首届“关汉卿戏剧文化节”，创排一批文艺精品。实施竞技体育设施提升工程、群众体育健身惠民工程，筹备好市第七届全民运动会，发展冰雪运动，做强体育产业，增强全民体质，打造健康保定。

维护安全稳定。持续深化平安保定、法治保定建设，当好首都政治护城河。加强乡村、社区等基层组织建设，推动社会管理重心下移。深入开展扫黑除恶专项斗争，坚持深挖彻查，“一案三查”，坚决铲除黑恶势力滋生土壤。加强应急保障工作，提高防灾减灾救灾水平。严格落实安全生产责任制，持续开展事故隐患大排查大整治，坚决遏制重特大安全事故。加大食品药品监管力度，开展药品、保健品违法营销专项整治，确保人民群众“舌尖上的安全”。同时，加强国防教育和国防后备力量建设，发挥工会、共青团、妇联等人民团体桥梁纽带作用，做好民族宗教、外事、人防、史志档案、气象、妇女儿童、老龄、残疾人等工作。

各位代表！在多渠道多层次征求群众意见的基础上，本着民生至上、积极而为的原则，今年拟在促进就业、教育提升、住房改造等11个方面办好49项惠民实事。我们一定坚持以人民为中心的发展思想，把人民群众身边的小事当作政府的大事，集中精力财力，坚决把实事办实、好事办好。

三、加强政府自身建设

“喊破嗓子，不如干出样子”。当前，保定发展机遇难得、也等不得、更耽误不得，我们有责任也有决心与全市人民一起拼搏、一起奋斗、一起实干，努力在实干中打造为民务实清廉的政府。

在实干中强化政治意识。旗帜鲜明讲政治，强化“四个意识”，落实“两个维护”。坚持干中学，切实学懂弄通做实习近平新时代中国特色社会主义思想和党的十九大精神，保持坚定的信仰、必胜的信心，永远听党的话、跟党走，把贯彻落实中央、省委省政府和市委的决策部署，作为检验讲政治的重要标尺，融入到思想中，体现在行动上。坚持学以致用、用以促学，增强“八种本领”，在深化改革扩大开放中不断解放思想，全面提升讲政治、识大局、定政策、促发展的能力。

在实干中严格依法行政。把政府工作全面纳入法治轨道，加强法治教育，运用法治思维推动工作，依靠法治方式解决问题，让法治精神成为全社会的共同追求。严格依法民主科学决策，严格规范执法行为，自觉接受人大法律监督、政协民主监督和社会舆论监督。抓实抓好政务公开，加强政策解读，主动回应社会关切，让权力在阳光下运行。

在实干中淬炼担当品质。强化担当履责、实干实绩导向，鼓励创新、宽容失败，增强正向激励，旗帜鲜明地为敢破敢立、敢闯敢干的干部鼓劲撑腰。整饬作风纪律，持续纠正“四风”，坚决反对和克服形式主义、官僚主义，坚决惩治庸政懒政怠政行为，坚决纠正损害群众利益的不正之风。持续开展大学习大调研活动，身子沉下去、服务提上来，贴近百姓、走进企业，民有所呼、必有所应，“居民吹哨社区报到、企业吹哨政府报到”，打造有感知、有温度的责任政府。

在实干中提升服务效能。坚持问题导向、突出实干实政，服务提质、工作提效，把解决问题、优质高效作为检验政府效能的重要标准。全面实施企业开办提速专项行动，持续深化“减证便民”行动，切实解决群众企业办事创业中的困难和问题，把该放的放开，该管的管好，让政府瘦身，给市场松绑，为百姓谋利。

在实干中恪守清廉本色。当好人民“勤务员”，既要干事成事，更要清白清廉。严守“八项规定”，落实“两个责任”，履行“一岗双责”，以永远在路上的坚韧和执着，全面加强政府系统党风廉政建设和反腐败工作，持之以恒正风肃纪。抓好中央和省委巡视反馈意见整改落实，着力解决审批监管、资源开发、国资国企、扶贫攻坚等重点领域和关键环节的腐败问题。严格执行廉洁自律准则和纪律处分条例，强化监督执纪问责，坚决查处违规违纪和失职渎职行为，打造一支忠诚干净担当的高素质公务员队伍。

各位代表！机遇不给空想者，行动造就实干家！让我们更加紧密地团结在以习近平同志为核心的党中央周围，在市委的坚强领导下，团结带领全市人民，提振精气神、跑出加速度，攻坚克难、乘势快上，为实现保定跨越、崛起和复兴，为建设经济强省、美丽河北作出新的更大贡献，以优异的成绩向中华人民共和国成立70周年献礼！
